# Simultaneous acoustic and photoacoustic microfluidic flow cytometry for label-free analysis

**DOI:** 10.1038/s41598-018-37771-5

**Published:** 2019-02-07

**Authors:** Vaskar Gnyawali, Eric M. Strohm, Jun-Zhi Wang, Scott S. H. Tsai, Michael C. Kolios

**Affiliations:** 1 Department of Mechanical and Industrial Engineering, Ryerson University, Toronto, Canada; 2 Department of Mechanical and Industrial Engineering, University of Toronto, Toronto, Canada; 3 Translational Biology and Engineering Program, Ted Rogers Centre for Heart Research, Toronto, Canada; 4 Department of Physics, Ryerson University, Toronto, Canada; 5 Institute for Biomedical Engineering, Science and Technology (iBEST), Toronto, Canada; 6 Keenan Research Centre, St. Michael’s Hospital, Toronto, Canada

## Abstract

We developed a label-free microfluidic acoustic flow cytometer (AFC) based on interleaved detection of ultrasound backscatter and photoacoustic waves from individual cells and particles flowing through a microfluidic channel. The AFC uses ultra-high frequency ultrasound, which has a center frequency of 375 MHz, corresponding to a wavelength of 4 *μ*m, and a nanosecondpulsed laser, to detect individual cells. We validate the AFC by using it to count different color polystyrene microparticles and comparing the results to data from fluorescence-activated cell sorting (FACS). We also identify and count red and white blood cells in a blood sample using the AFC, and observe an excellent agreement with results obtained from FACS. This new label-free, non-destructive technique enables rapid and multi-parametric studies of individual cells of a large heterogeneous population using parameters such as ultrasound backscatter, optical absorption, and physical properties, for cell counting and sizing in biomedical and diagnostics applications.

## Introduction

For many decades, flow cytometry has been one of the most effective and powerful approaches for rapidly analyzing the characteristics of single-cells in a large population^1–3^. Flow cytometers use either optical or electrical impedance based techniques to analyze cell populations^4–6^. In optical-based techniques, signal sources come from side and forward scattering of light from suspended individual cells, and fluorescence emission from intracellular components tagged with fluorescent molecules^7^. When the cells flow through an interaction zone, they interact with a laser beam, which causes the generation of the signals^8^. Forward scattered light is associated with cell size, side scattered light correlates with the structural complexity of the cells, and fluorescence signals relate to the type of intracellular components in the cells^1^. In impedance-based flow cytometers, the electrical impedance changes when a cell passes through an electric field. This change is correlated with the volume of the cell^9–11^.

Despite being ubiquitous in biomedical research, these classical flow cytometry approaches have several limitations. On one hand, light scattering from cells in optical-based cytometers provide information limited to cell size and granularity^8^. To detect DNA or RNA content, proteins, or antigens, typically fluorescent dyes or fluorescence-tagged antibodies are attached to either specific intracellular components or to the cell membrane. The fluorescent signal can then be analyzed to extract the information of the cellular state by the detection of molecules or intracellular components to which the fluorescent molecules are attached^12^. Some fluorescent markers can be cytotoxic^13^. Moreover, the quality of the data generated when using fluorescent probes critically depends on sample preparation^14^. For some applications, such as gene expression and protein localization, higher fluorescence expression is required to achieve sufficient sensitivity^15,16^. On the other hand, impedance-based systems are only able to count and size cells and do not provide information of cell constituents^17^. Due to these limitations, there is ongoing research in developing new flow cytometry approaches.

A candidate technique for implementation in flow cytometry involves ultrasound scattering, which has demonstrated utility in analyzing cells^18–21^. Acoustic waves above a frequency of 100 MHz are highly sensitive to cell physical properties, such as shape and size, and biomechanical properties, such as Young’s modulus^22,23^. Additionally, as a response to optical irradiation, cells may generate photoacoustic (PA) waves, which are dependent on the optical absorption properties of the cell and its constituents^24,25^. Thus, US and laser pulses can interact with a particle or cell to generate two distinct mechanical waves: US backscatter and PA waves^22,23,25,26^. The former results from the acoustic impedance mismatch between the particle and the surrounding medium^27–29^. The latter results from the optical absorption of the particle^24,30^, which generates a rapid thermoelastic expansion that creates broadband mechanical waves with frequencies in the ultra-high frequency (UHF) range. The generation and characterization of US backscatter and PA waves from solid and liquid spheres are well-described^26,31^. This theoretical framework can therefore be used in experimental measurements to infer properties of nearly spherical objects, such as cells. For example, various attempts have been reported in the literature to study flowing cells using photoacoustics *in vivo*. Namely, Zharov *et al*. used gold nanorods as contrast agents tagged to tumor cells to detect the tumor cells using photoacoustics^32,33^, He *et al*. used high-resolution imaging to detect circulating melanoma cells^34^ and target the cells with lethal irradiation using a therapy laser^35^. Similarly, Ning *et al*. used photoacoustic microscopy at ultrasound frequency (35 MHz) to map *in vivo* blood vessel diameters, *sO*_2_, blood flow, and resolve individual red blood cells in the capillaries, by analyzing photoacoustic microscopic images^36^. All of these *in vivo* examples demonstrate the utility of US and PA in cellular analysis. However, the above techniques could not be used to size the cells.

Combining US backscatter and PA in single cell analysis *in-vitro* enables label-free and multi-parametric analyses based on two different physical interactions^37^. On one hand, unique US backscatter signatures from individual cells provide information on cell size, shape, structure, and biomechanical properties^38,39^. Existing reports of single cell analysis using acoustic microscopy demonstrate the capability to extract cellular properties^28,40^. On the other hand, PA signals contain information about the intracellular components that absorb the optical irradiation energy such as lipids, mitochondria, and deoxyribonucleic acid (DNA)^41,42^. The source of the PA signal depends on the laser wavelength used and presence of the optically absorbing structures at those wavelengths. Since an ultrasound transducer is used to detect both US and PA waves, both US and PA can be used to probe the same cell^43^. However, in static microscopy systems the throughput is very low, (typically on the order of a cell per minute)^20,21^. There is therefore an unmet need for a label-free technique that rapidly probes suspended individual cells without the limitations of conventional flow cytometry approaches, such as the requirement of fluorescent-tagging and lack of a method to rapidly probe biomechanical cell properties.

Here, we present a label-free and high throughput flow cytometry technique by combining UHF US backscatter and laser-induced PA waves generated from the same individual cells or particles in a microfluidic platform. Our technique builds upon previous combined US and PA static methodology^25^, by integrating with microfluidics, to achieve high throughput label-free analysis of single cells in heterogeneous solutions. We first use this acoustic flow cytometer to differentiate and count different color polystyrene microparticles, and we find results consistent with those obtained from a commercial fluorescence-activated cell sorting (FACS) system. We then show that this acoustic flow cytometer can identify and count red blood cells (RBCs) and white blood cells (WBCs), again showing good agreement with results from the commercial FACS system.

The main advantage of this approach is that multiple biophysical parameters are acquired simultaneously, label-free, from the same single cell. This is achieved by integrating an US transducer to a polydimethylsiloxane (PDMS)-based microfluidic device to detect both US and PA signals emitted from particles or cells flowing inside the microfluidic channel. The detection of US backscatter and a PA signal provides multiple biophysical characteristics of the same cell. Physical and morphological properties are derived from the US backscatter and optical absorption properties are derived from the PA waves. The approach of probing the same particle with both US and PA limits the possibility of environmental and time-dependent experimental variations of the cells probed. Moreover, by targeting distinct endogenous chromophores using different optical excitation wavelengths with a tunable laser (e.g. UV laser excitation to target DNA/RNA), different intracellular components can be detected, and in principle, quantified. The same methodology can be used for the characterization of extracellular vesicles (EV) and other biological specimens of interest which we are currently pursuing.

Our label-free multiparametric approach also has advantages in sample preparation. When analyzing blood samples using optical-based flow cytometry methods, WBCs are isolated with density gradient separation. The resulting RBCs are lysed before tagging the WBCs with fluorescent molecules. This is done to minimize the interference of signals produced by various cells in the sample^44,45^ and is important in immunophenotyping peripheral blood T-cells, as well as and their subsets, for diagnostic applications^46^. Additionally, the RBC lysis buffer increases the membrane permeability and alters the plasma membrane, damaging WBCs. RBC lysis can also cause leukocyte activation^47–50^. In the acoustic flow cytometry approach we propose, WBCs and RBCs can be easily differentiated based on their optical absorption. Therefore, the adverse effects of buffers and reagents on the WBCs can be avoided using acoustic flow cytometry. Furthermore, our approach does not need expensive antibodies and fluorescent molecules, lowering the overall cost of the test^51^.

Further processing of the US and PA signals from flowing cells has potential applications in investigating the biomechanical and optical absorption related properties of individual cells^52^. Therefore, the proposed acoustic flow cytometry approach can be used in a variety of biological applications that are based on counting, sizing, and identifying cells in a sample, for example, in detecting and quantifying circulating tumor cells in blood.

## Results

### Acoustic flow cytometry concept

**Figure 1:**
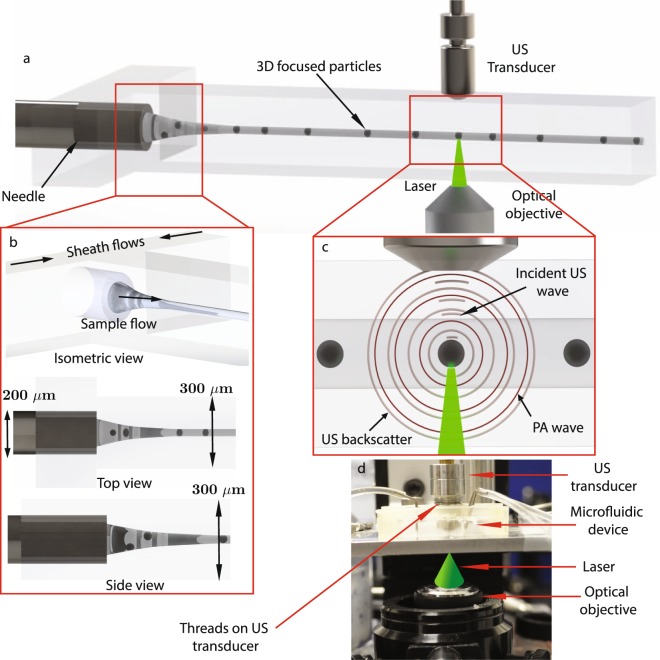
Conceptual schematic illustration of the acoustic flow cytometry system. (**a**) The illustration shows the overall design of a microfluidic device with collinearly aligned US transducer and laser focusing optical objective. (**b**) Hydrodynamic 3D flow focusing of a sample flow in the microfluidic device. The isometric view shows how the sample flow infused through the embedded needle at the cross-junction is 3D focused by the sheath flows from the side channels. The top and side views illustrate the position of the needle relative to the channel geometry. Gaps above, below, and on the sides of the needle allow the sheath flows to push the sample flow to the center of the channel. (**c**) The magnified view of a collinearly aligned transducer and laser beam. The incident US wave and laser beam interact with a particle at the interrogation zone producing both US backscatter and PA waves. (**d**) Image of an experimental setup that shows how the laser is focused on the interrogation zone of the US transducer.

The acoustic flow cytometer is composed of a microfluidic device and fluid manipulation pumps; an ultrasonic system consisting of an UHF transducer, ultrasonic pulse generator, and control tools; and an optical system consisting of a laser source and optical components such as lenses, mirrors, and light sources. The conceptual illustrations of the acoustic flow cytometer in Fig. 1 shows how the microfluidic, ultrasonic, and optical systems are integrated. In this system, a heterogeneous suspension of particles or cells is hydrodynamically focused (Fig. 1a,b), and passes through the focal zone of the US beam and focal spot of the laser (i.e. interrogation zone), generating the US backscatter and PA waves (Fig. 1c).

### Generation of ultrasound backscatter and photoacoustic signals

**Figure 2:**
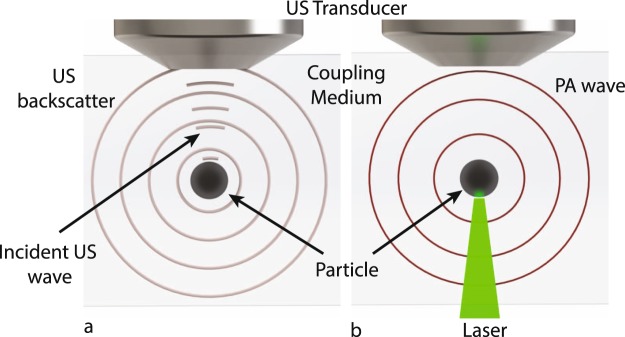
(**a**) the ultrasound (US) backscatter and (**b**) the photoacoustic (PA) waves that are generated by a particle located in the focal zone of the transducer, as a result of the ultrasound and laser pulses that interact with the particle, respectively.

**Figure 3:**
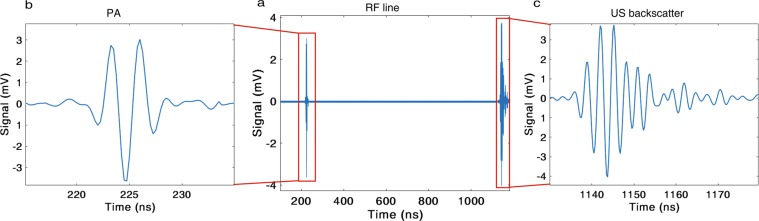
(**a**) A representative RF line detected and recorded by the ultrasound (US) transducer from a 3 *μ*m diameter black polystyrene particle. The ultrasound and photoacoustic (PA) signals in the RF line are highlighted in the red boxes. Transducer artifacts are removed from the signal, by subtracting background signals, to emphasize the detected ultrasound backscatter and photoacoustic waves. This background subtraction is common in acoustic microscopy^28^. Measured ultrasound backscatter (**c**) and photoacoustic (**b**) signals from the individual particle. These signals are gated using hamming window from the RF line.

Figure 2 shows schematic illustrations of the US backscatter and PA waves generated by a particle located in the focal zone of the transducer. An incident US pulse scattered by the particle is detected as US backscatter (Fig. 2a). Similarly, the optical energy absorbed by the particle generates PA waves (Fig. 2b). In our device, the laser and US transducer are triggered simultaneously. The speed of light is much faster than the speed of sound, thus the laser excites the particle first, generating a PA wave. Approximately 900 ns later, the US pulse scatters off the particle. The PA wave is detected first while the reflected US is detected by a short delay. Fig. 3 shows a typical RF line measured from a 3 *μ*m black polystyrene bead (processed so that the transducer internal reflections are subtracted). To analyze the detected signals, the individual PA and US signals are isolated using a Hamming window and saved for further processing.

### Calibration of the acoustic flow system

Black polystyrene particles have high optical absorption, in contrast to white particles. Hence, black particles produce both US and PA waves, while white particles produce only US backscatter that is strong enough to be detected by the transducer at the optical wavelengths used.

**Figure 4:**
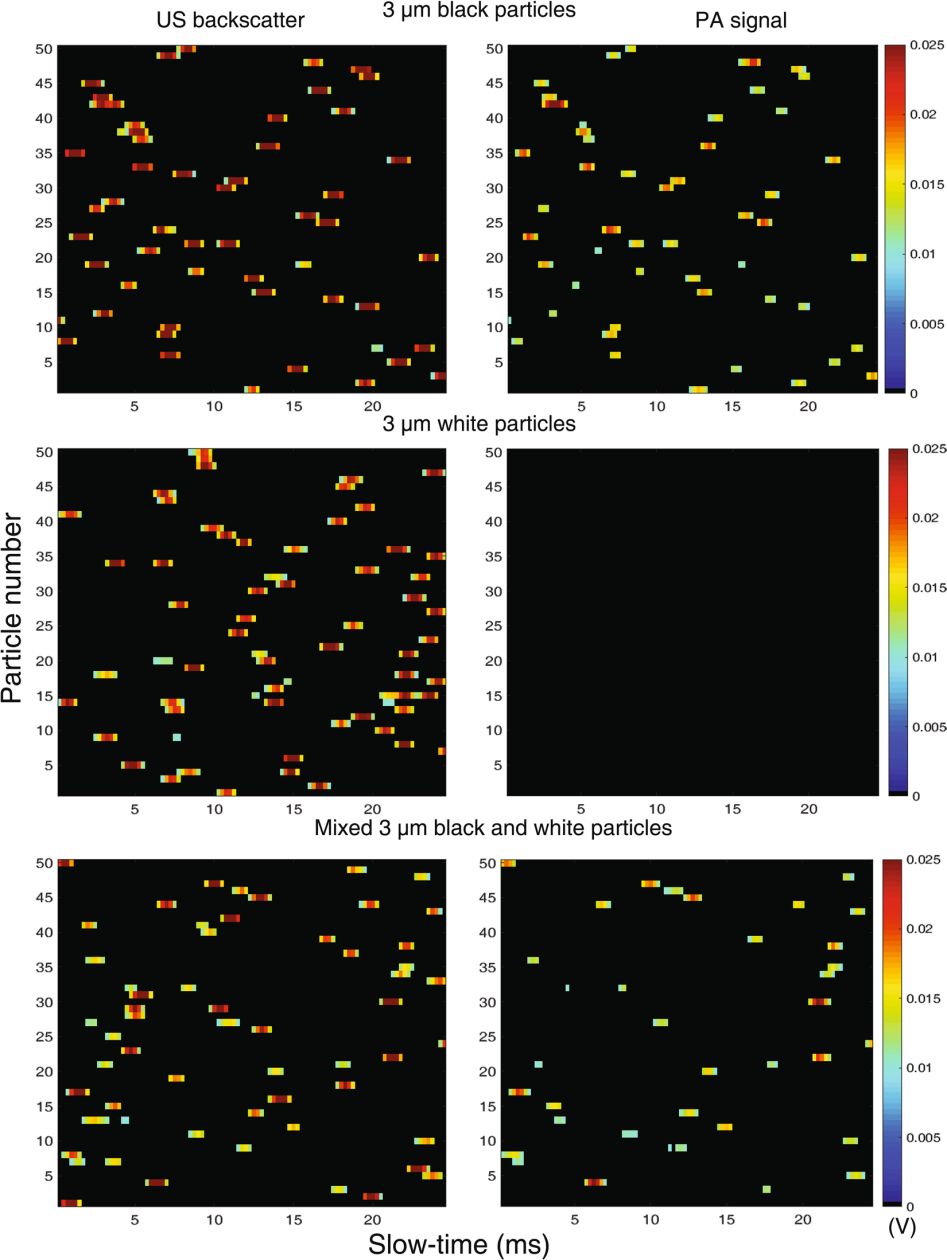
Maximum amplitude plot of the time domain signals of the US backscatter and PA waves from individual particles as detected by the transducer for three different samples: black particles exclusively (top row), white particles exclusively (middle row), and a mixture of both types of particles (bottom row). The y-axes represent 50 particles detected by the acoustic flow cytometer from each experiment. The x-axes represent the “slow-time” of the acquisition by the transducer while the particles flow downstream. The amplitude of the detected signals (in V) is represented by the color bars.

For each particle detected by the system, we record one hundred subsequent RF lines at a pulse repetition frequency 4 KHz (referred as the slow-time) and each signal is sampled at 4 GHz sampling rate (referred as the fast-time). The concept of fast and slow-time is shown in SI, Fig. S1. As the particles pass through the interrogation zone, RF lines containing the US and PA signals are recorded as the particles flow through the channel. Fig. 4 shows plots of the maximum amplitude of the US backscatter (left column) and PA (right column) signals detected from individual experiments using black particles exclusively (top row), white particles exclusively (middle row), and a mixture of both black and white particles (bottom row). Color intensity represents the maximum amplitude of the acquired RF signals. The y-axis represents a number that denotes consecutive acquisitions for which a signal (whether US or PA) is detected. The x-axis shows the slow-time of signal acquisition. The results from the experiments show that for particles (both black and white) passing through the interrogation zone for which an US backscatter signal was detected, only black particles produce PA signals. The top row in Fig. 4 demonstrates that almost 100% of the detected black beads produce both the US and PA signals. The middle row (white beads) contains only US signals, and the bottom row (mixture) contains US and some PA signals.

### Comparison with Fluorescence Activated Cell Sorting (FACS)

**Figure 5:**
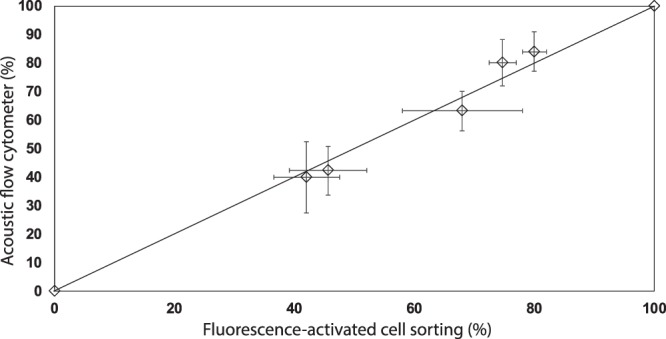
A comparison plot of the percentage of black particles in sample solutions of a mixture of black and white particles detected by the acoustic flow cytometer and FACS. Results from the acoustic flow cytometer are comparable with those from FACS. Error bars represent one standard deviation with data from five independent experiments. The straight line is the line of equality.

We compare results from the acoustic flow cytometer to those from FACS using the same sample solution of 3 *μ*m polystyrene black and white particles. The auto-fluorescence signal produced by white particles is significantly less than the signals from black particles in the APC and PE-Cy5 channels. The autofluorescence information is used to differentiate the particles. See the SI (Fig. S2) for more detailed information about the autofluorescence of the polystyrene particles. To compare the results from both cytometry systems, we use mixtures of the black and white particles at five different volume ratios taken from stock solutions of black and white particles. For each volume ratio, we perform five experiments in both the acoustic flow cytometer and FACS. The results of five experiments of each sample solution in both systems are averaged and plotted in the graph shown in Fig. 5. The *y*-axis represents the percentage of the black particles in the solution detected by the acoustic flow cytometer whereas the *x*-axis is the percentage of black particles detected by FACS. The close proximity of the data points to the solid straight line in the graph shows that the measurements of the acoustic flow cytometer are in close agreement with those from FACS (*R*^2^ = 0.9881). Error bars represent one standard deviation from the five experiments performed on FACS and acoustic flow cytometer, respectively. For each sample, we measure at least 500 particles using the acoustic system and 10,000 particles using FACS. The data points at 0% and 100% represent the samples with only white particles and black particles, respectively. In both cases, the acoustic flow cytometry system correctly identified all particles.

### Label-free blood cell differentiation

We experiment with human blood to demonstrate the acoustic flow cytometer’s ability to differentiate between red and white blood cells in a label-free manner. RBCs absorb light at 532 nm, generating strong PA signals, whereas WBCs do not significantly absorb light at this wavelength^53^. Therefore, WBCs do not generate detectable PA signals. Individual RBCs produce both US backscatter and PA signals, whereas WBCs produce only US backscatter. The PA signal from the WBC is not strong enough to be detected by the transducer at the optical wavelengths used due to the absence of strongly absorbing structures. This is the principle with which the acoustic flow cytometer differentiates between WBCs and RBCs.

**Figure 6:**
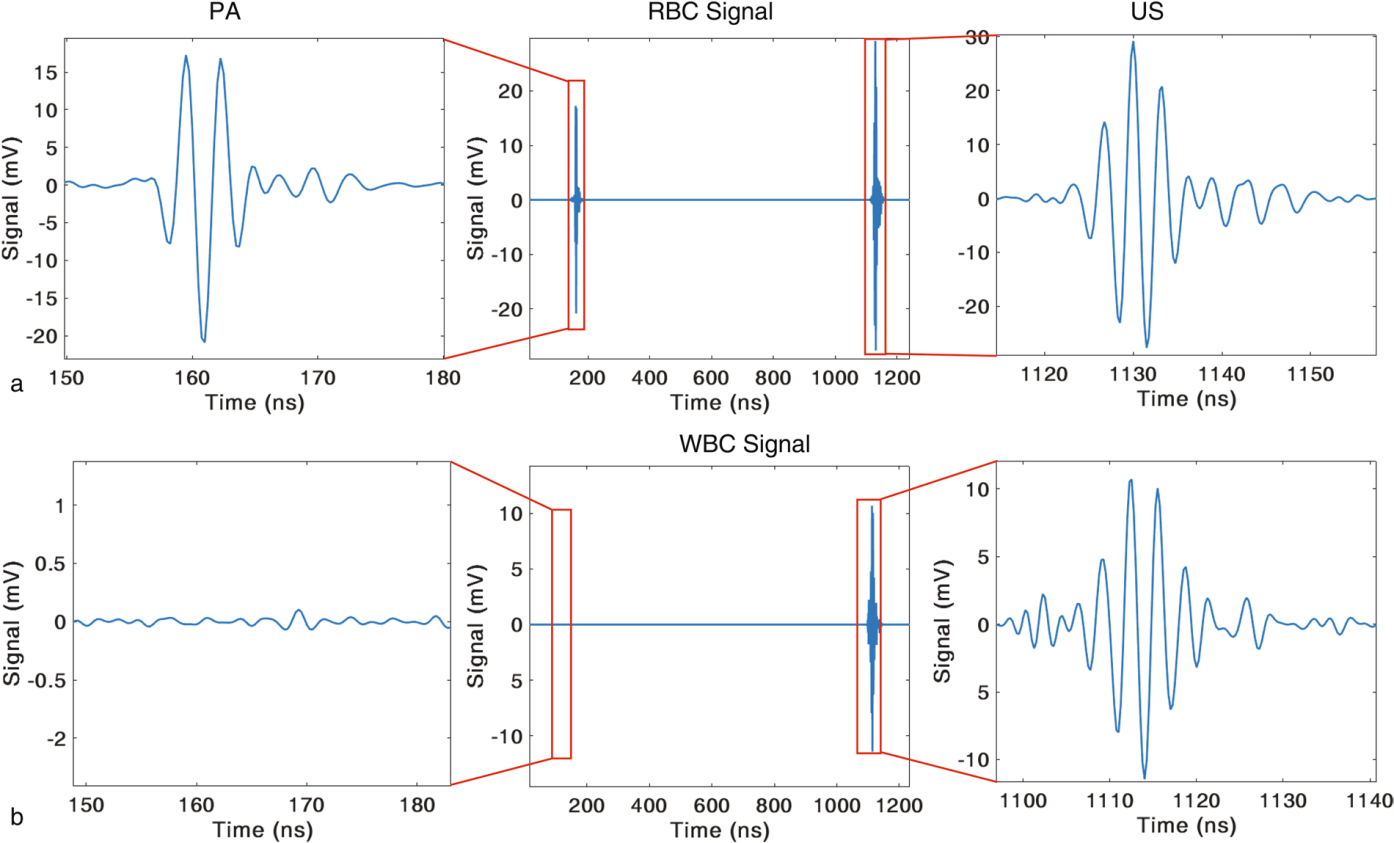
Representative signals detected by the transducer for (**a**) RBC and (**b**) WBC samples. The signal from a RBC contains both US backscatter and PA waves whereas signals from a WBC contains only US backscatter.

We use sample solutions of isolated WBCs. These sample solutions contain various concentrations of residual RBCs along with the isolated WBCs without platelets. By evaluating the presence or absence of a PA signal, we classify the source of the signal as either a RBC (i.e. when US and PA signals are both present) or a WBC (i.e. when only US signals are present). Using all the RF lines with signals detected, we calculate the percentage of RBCs and WBCs present in the sample solutions. Representative experimental RF lines from both individual RBC and WBC are shown in the Fig. 6a,b, respectively.

We compare these results to those obtained with FACS. Here, we separately incubate two aliquots of 200 *μ*L of each sample solution with DRAQ5 fluorescent dye and R-PE conjugated CD45 antibodies to label the cells. The DRAQ5 stains the nuclei of the WBCs while the CD45 antibodies attach to the membrane of the WBCs in the solutions, whereas neither DRAQ5 nor CD45 interact with RBCs. We dilute these solutions in phosphate-buffered saline (PBS) solution before introducing them into FACS. Based on the detected fluorescence signals from the sample solutions, we calculate the percentage of RBCs and WBCs from both experiments.

**Figure 7:**
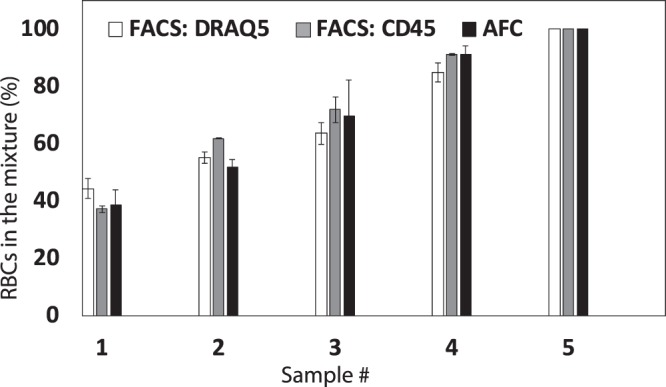
Plot showing the percentage of RBCs in mixtures of RBCs and WBCs from five sample solutions containing WBCs and RBCs using the acoustic flow cytometer (labeled AFC) and FACS. The y-axis represents the percentage of the RBCs present in the sample solutions. For each experiment, the sample solutions are aliquot to three different volumes. The first volume is label-free, the second is incubated with DRAQ5 fluorescent dye, and the third is incubated with R-PE conjugated CD45. DRAQ5 stains WBC nuclei and CD45 bonds to the WBC membrane. The label-free solution is measured using the AFC, and the other two are measured using FACS. Error bars represent one standard deviation from the mean for three experiments.

We experiment using five different sample solutions, each containing different concentrations of RBCs and WBCs, to compare the acoustic flow cytometer and FACS. The sample numbers are shown on the *x*-axis in Fig. 7, and the *y*-axis shows the measured percentage of RBCs. We test each sample three times and the average concentration for each cell type is plotted on the *y*-axis. The black bars represent results from label-free acoustic flow cytometry, grey bars show measurements from CD45 tagged WBCs using FACS (FACS:CD45), and white bars show data from nuclei stained WBCs using FACS (FACS:DRAQ5). For each acoustic flow cytometer experiment, we measure at least 500 individual cells while for each FACS experiment, we measure approximately 10,000 cells. Error bars represent the standard deviation of the mean for three experiments. This figure shows good agreement between the results obtained from the acoustic flow cytometer and FACS.

## Discussion

In summary, we demonstrate a label-free cytometry technique based on acoustic waves to analyze individual cells in a heterogeneous population. The same cell is probed by UHF US, with a center frequency of 375 MHz, and a 532 nm nanosecond laser. We show that the acoustic flow cytometer is able to differentiate between WBCs and RBCs in a label-free manner, and the measurements show good agreement with the results obtained from FACS using labeled samples. To the best of our knowledge, this is the first demonstration of using US backscatter and PA waves in a high throughput microfluidic platform for the flow cytometry of individual cells. The current version of the acoustic flow cytometer has a throughput of 150 cells per minute. The throughput is primarily limited by the repetition frequency of the common trigger for the ultrasound and laser pulses (4.1 kHz), and the duty cycle of the digitizer used in the system. The pulse repetition frequency is controlled by trigger signals synced with the output trigger signal generated by the laser source. This limitation can be overcome by using a separate trigger source with higher repetition frequency. The latency time of the current digitizer (Cobramax, Gage Applied, USA) is in the range of a millisecond, which also lowers the effective pulse repetition frequency of the US/PA laser. Upgrading the digitizer system will help improve the throughput of the flow system. Similarly, the throughput of the acoustic system can also be further improved by optimizing the microfluidic device. For example, better flow focusing of the individual cells can be used to ensure that they are in the focal zone of the transducer. The velocity of cells can be changed to better synchronize detection events with the repetition frequency of the US and laser pulses^54^.

For this flow cytometer with confocal ultrasound and light, precise control and focusing of the sample particles is crucial. For our flow cytometry system, we developed a needle-based flow focusing technique, which provides stable and precise 3D hydrodynamic focusing of sample flow. Suspended individual particles and cells flow in a single stream through the interrogation zone. The 3D flow focusing is important to maintain a gap between the sample cell and the surface of the glass substrate. This separation avoids the overlap of the signals between US backscatter from the cell and US reflection from the substrate. In addition, this simple 3D flow focusing technique developed using a single-layered channel dimension can be used as an alternative for microfluidic applications, such as droplet generation^55^.

The US backscatter and PA signals detected from individual cells can be used to extract information regarding the physical properties of cells, such as size, shape and the optical absorption properties of the cell or its intracellular components. We previously showed that US backscatter alone can be used to size individual cells in either stationary^43^, or in microfluidic flow systems^56^. However, no discrimination based on optical properties can be achieved with this system and it is not trivial to distinguish between RBCs and WBCs based on US scattering alone. This opens many possibilities for cell differentiation and classification. Similarly, with the addition of PA, the optical absorption properties of the cell components can be correlated with functional properties of cells. In RBCs, we can estimate the oxygen saturation or hemoglobin concentration in an individual cell. Analysis of the RF signals (Figs 3 and 6) can also provide information related to the structures of that scatter ultrasound, and the structures that absorb the optical energy^43,57^. However, there are biological safety threshold limits of the laser fluence on cells at both UV (3 *mJ*/*cm*^2^ for wavelengths between 180–302 nm) and visible wavelength range (~500 *mJ*/*cm*^2^)^58,59^. If cell viability is required in the application, our system must overcome the challenge of maintaining the laser threshold at different wavelengths and still maintain an adequate signal to noise ratio of the PA signals detected from individual cells. One way to address this is to use higher frequency US transducers, in the range of GHz, to increase the detection limit while using the laser fluence within the threshold limit. Despite the greater ultrasound attenuation at these frequencies, PA signals from smaller structures are expected to have a greater amplitude^57^. With these applications, we believe that the acoustic flow cytometry technique can provide unique advantages compared to optical-based techniques. Furthermore, acoustic flow cytometry may find utility in applications where label-free identification is important, such as the identification of different cell types and circulating tumor cells and *in vivo* cell analysis.

## Methods

### Microfluidic device fabrication

To fabricate an integrated microfluidic device, we use a 3D printed (ProJet 3510 HD, 3D systems, Rock Hill, SC, USA) transducer mold (see SI, Fig. S3a), a 3D printed alignment frame (Fig. S3c), and a CNC machined metal mold (Fig. S1d). The transducer mold, which is a replica of the US transducer (Fig. S3a), retains the dimensions and the screw threads of the transducer. The tip of the transducer mold contains a hole of 300 *μ*m in diameter and 2 mm deep. The metal mold contains a 1 mm tall and 280 *μ*m diameter vertical pin, on the channel structure positioned next to the cross junction (Fig. S3d). For making the final microfluidic device, we first screw the transducer mold into the alignment frame and inserted both onto the metal mold ensuring that the pin slides into the hole. (An arrow is used to indicate the hole and the pin in Fig. S3). The assembly then is filled with liquid PDMS and stored in an oven at 70 °C for 3 hours. Afterwards, the cured PDMS chip along with the 3D printed molds (Fig. S3e) are removed from the metal mold, the transducer mold is unscrewed, and the resulting chip is bonded onto a microscope slide using oxygen plasma (Harrick Plasma, Ithaca, NY, USA). A nanoneedle is inserted into the center of the cross junction and is glued to the glass slide using two-component epoxy glue (Henkel Canada Corporation, Mississauga, ON, Canada). Finally, the US transducer is screwed into the device (Fig. S3f). The pin-hole design on the molds automatically aligns the transducer to the channel (Fig. S3g) and opens an interconnection between the transducer and the microfluidic channel.

We use a 375 MHz single element focusing UHF US transducer (Kibero Gmbh, Germany), which has a focal length of 300 *μ*m. The focal zone has an ellipsoidal geometry with major and minor axes of 15–20 *μ*m and 5–8 *μ*m, respectively. Since sample cells need to flow through this small focal zone, in a single stream, we require a precise and controlled method to focus the sample solution. To achieve this, we modify a stable two dimensional (2D) flow focusing microfluidic setup we reported previously for this application^60^. Namely, we design our PDMS (Sylgard 184 silicone elastomer kit, Dow Corning, Midland, MI, USA) based microfluidic device with a cross-junction channel architecture. Figure 1b shows that at the junction, sample flow injected through an integrated nanoneedle (ID = 100 *μ*m, OD = 200 *μ*m, Japan Bio Products Co. Ltd., Tokyo, Japan) meets the two sheath flows. At the cross-junction (Fig. 1b, isometric view), a needle-guiding channel, two side channels, and a downstream channel meet, which are 100, 200 and 300 *μ*m wide, respectively. This geometry enables the sheath flow to hydrodynamically focus the sample flow in both the lateral and axial directions.

Another critical aspect for our microfluidic acoustic flow cytometer is the integration and alignment of the US transducer with the focused flow in the microchannel (Fig. 1c). To achieve the precise alignment of the transducer, we exploit the threaded cylindrical surface of the US transducer, and mold similar threads to the transducer cavity in our microfluidic mold (see threaded transducer surface in Fig. 1d). After the PDMS microfluidic device is patterned, the US transducer is manually inserted into the cavity of the PDMS microfluidic device. The lateral alignment of the transducer with the focused flow is further refined by adjusting the flow rates of the sheath flows, and the axial alignment is achieved by moving the transducer in or out of the threaded cavity. We use a Pump 11 Elite syringe pump (Harvard Apparatus, Holliston, MA, USA) to infuse the sample flow (0.5–5 *μ*L/min) and a pressure pump (CorSolutions LLC., Ithaca, NY, USA) to infuse the sheath flows (15–60 *μ*L/min). We use polystyrene microparticles (Polysciences Inc., Warrington, PA, USA) for alignment experiments because they produce strong backscatter signals, and we adjust the sheath flow and transducer axial position to achieve maximum backscattered amplitude signals, indicating good alignment. For PA signals, the laser (Teem Photonics Inc., France) spot is focused by adjusting the device using a mechanical X, Y, Z-stage to ensure that the transducer and the laser foci are collinear (Fig. 1a).

### Ultrasonic and optical systems

A PCI card-based trigger source triggers the US and the laser pulses. Monocycle pulses from a 300 MHz pulser produce US pulses from a single element focus US transducer with a center frequency of 375 MHz. The focal length of the transducer is 300 *μ*m and the focal zone is an ellipsoid with lateral and axial semi-axes of 15–18 *μ*m and 5–8 *μ*m, respectively. Signals detected from the transducer are high-pass filtered (>100 MHz) and amplified by 30 dB (Miteq, USA). Resulting signals are then acquired by a Cobramax digitizer card (Gage Applied, USA) connected to a computer.

Laser pulses pass through a beam expander and an optical fiber before being reflected, from a combination of mirrors, towards a dichroic mirror. The dichroic mirror selectively reflects the 532 nm laser (pulse energy 20–50 nJ/pulse) to a 10X optical objective that focuses the laser beam to the interrogation zone. A xiQ CCD camera (Ximea GmbH, Germany) is used to monitor inside microchannel during the experiments. A detailed flow diagram of the acoustic flow cytometry system is presented in Fig. S4 of the SI.

### Sample preparation

Two different stock solutions of 3 *μ*m diameter black and white polystyrene particles (Polysciences, USA) in deionized (DI) water were prepared at a concentration of 6–7 × 10^6^ particles/mL. The particles were added to DI water and vortexed for homogeneous mixing. The stock solutions were vortexed every time before they were used.

A unit of whole blood was obtained from the Canadian Blood Services. WBCs from the whole blood are isolated using Histopaque-1077 (Sigma-Aldrich Corporation, St. Louis, MO, USA). Briefly, an equal volume of blood was used to form a double gradient over the Histopaque-1077, followed by centrifugation at 400 x g for 30 min and buffy coat containing WBCs was removed and diluted in PBS solution. The isolated WBC solution contains a number of residual RBCs. The isolated solution containing both RBCs and WBCs is diluted to approximately 4–6 million cells per mL in PBS and is passed through the acoustic flow cytometer. From the diluted solution, we take one aliquot of 200 *μ*L that is incubated with CD45 antibodies (Human CD45, R-PE conjugate, Life Technologies, Carlsbad, CA, USA) for 15 mins, and another aliquot of 200 *μ*L that is incubated with DRAQ5 (Thermo Fisher Scientific, Waltham, MA, USA) for 30 mins. The CD45 binds only to WBCs and DRAQ5 is a supravital fluorescent dye that stains WBC nuclei. These CD45 tagged and DRAQ5 stained samples are used in FACS (BD LSRFortessa X-20, Becton, Dickinson and Company, Franklin Lakes, NJ, USA) experiments.

### Signal analysis

The signals were processed after the complete acquisition. During the acquisition, RF lines from a cell or particle were saved if the US signals were higher than a threshold that indicated adequate SNR and that depends on the transducer used (3.5 mV in this work). Every saved signal was then separately windowed and a band pass filter was used to see if it contains both US and PA signals. In case of particles, if RF lines contained both the US and PA they were counted as signals from a black bead. Otherwise, they were counted as white beads. Similarly, for blood cell experiments, the signals containing both US and PA were counted for RBCs and only with US were counted as WBCs.

## Conclusion

We develop a label-free acoustics based microfluidic flow cytometer based on interleaved US backscatter and PA waves detected from individual cells or particles flowing through a microfluidic channel. The sample flow is hydrodynamically focused at a cross junction microchannel when flowing through a nanoneedle in a microfluidic device. We use high-frequency US backscatter (center frequency 375 MHz) and 532 nm pulsed laser-induced PA signals to analyze individual cells.

We calibrate the system using 3 *μ*m polystyrene particles. Our system is capable of differentiating the particles of various colors using the absorption property of the particles. The results obtained from the acoustic flow cytometer is in agreement with those obtained from FACS. In addition, we also show that our system is capable of label-free differentiation of RBCs from WBCs, and the measurements from the system are consistent with the results from FACS. This acoustic flow cytometer has potential applications in label-free cytometry to count, size, and identify individual cells from a cell population. It may also find future utility in the detection of circulating tumor cells and performing single cell characterization of flowing cells for diagnostic applications using blood or other bodily fluids.

## Supplementary information

Supplementary file

## Data Availability

The datasets generated during the current study are available from the corresponding author on reasonable request.
