# Supplementary material for: Simultaneous acoustic and photoacoustic microfluidic flow cytometry for label-free analysis
Source: Sci Rep. 2019 Feb 7;9:1585. doi: 10.1038/s41598-018-37771-5 (PMC6367457; doi:10.1038/s41598-018-37771-5)
Supplement: Supplementary file 1 — Supplementary file [file 41598_2018_37771_MOESM1_ESM.pdf]

Supplementary information

Simultaneous acoustic and photoacoustic microfluidic flow cytometry for label-free analysis

Vaskar Gnyawali,[1,5,6] Eric M. Strohm,[2,3] Jun-Zhi Wang[5,6], Scott S. H. Tsai,[1,5,6] Michael C. Kolios [4,5,6*]

[1]Department of Mechanical and Industrial Engineering, Ryerson University, Toronto, Canada, [2]Department of Mechanical and Industrial Engineering, University of Toronto, Toronto, Canada, [3]Translational Biology and Engineering Program, Ted Rogers Centre for Heart Research, Toronto, Canada, [4]Department of Physics, Ryerson University, Toronto, Canada, [5]Institute for Biomedical Engineering, Science and Technology (iBEST), Toronto, Canada, [6]Keenan Research Centre, St. Michael's Hospital, Toronto, Canada

Slow and fast-time signals

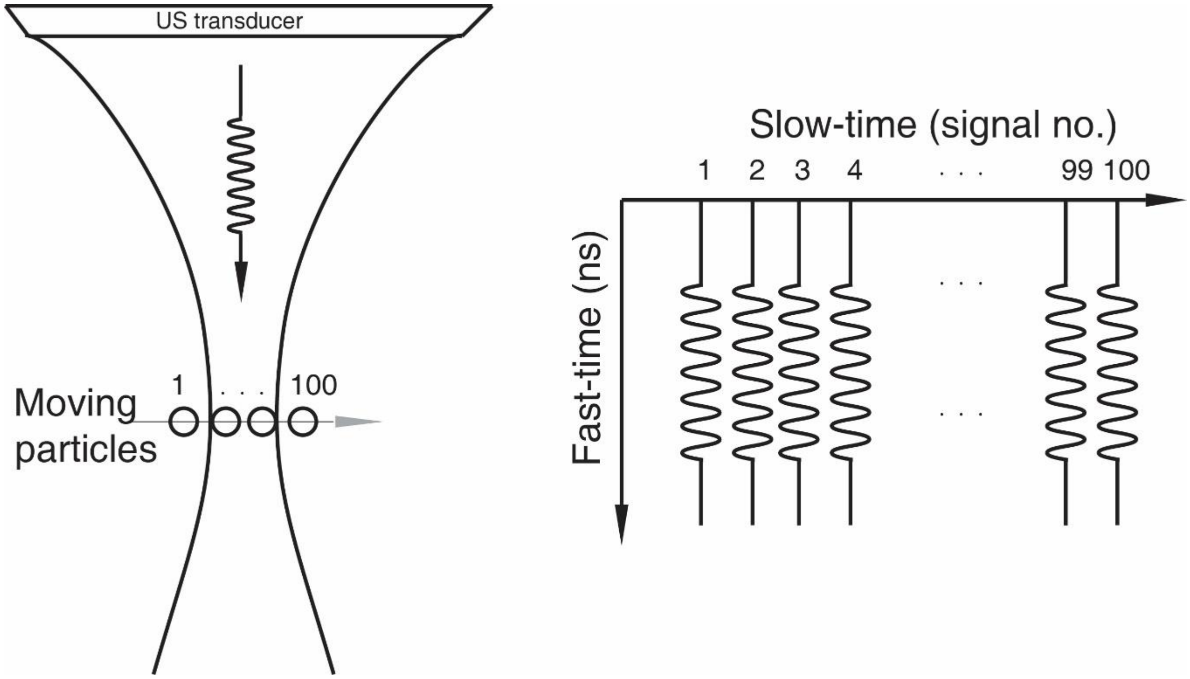

Figure S1. The schematic illustration of slow and fast-time axes representing signals detected by the US transducer while a particle flows through the focal zone of the transducer.

Autofluorescence detected from polystyrene particles using FACS

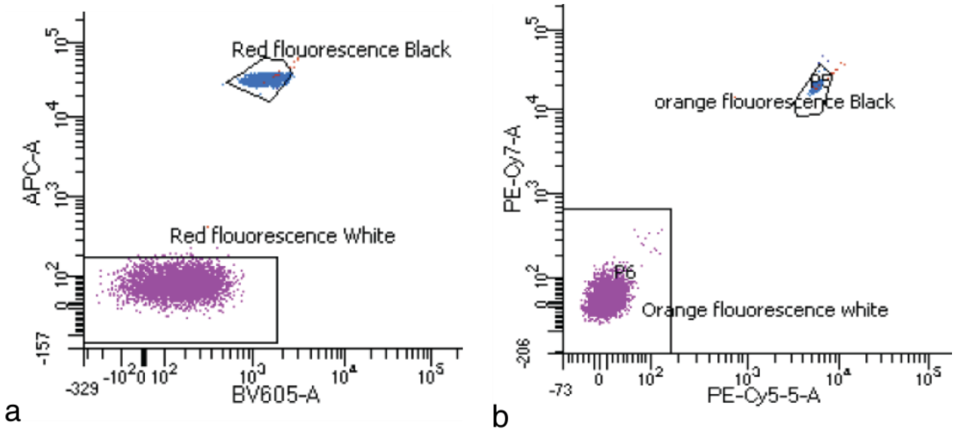

Figure S2. FACS results from 3 µm diameter polystyrene black and white particles, which are denoted as black and white, respectively. Data detected in the APC and BV605 channels (a) and PE-Cy7 and PE Cy5 channels (b) for the same sample of particles.

The sample contained a mixture of black and white polystyrene particles of 3 µm diameter. The autofluorescence signals produced by white and black particles at different channels, namely APC, BV605, Cy5, and Cy7, are combined to distinctly separate black particles from white particles using the FACS system. This allowed the comparison with the results from the AFC

Microfluidic device fabrication

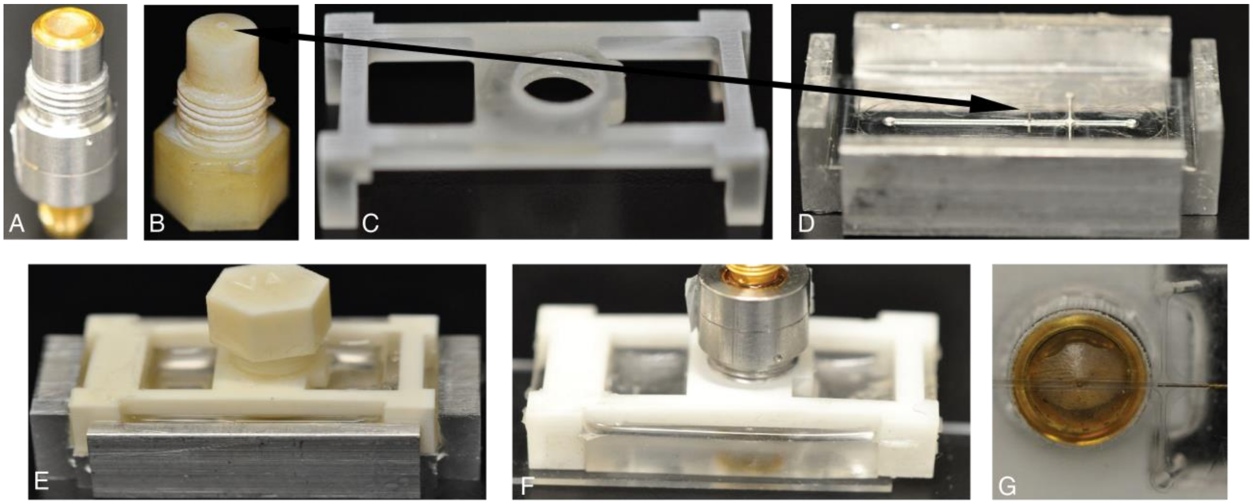

Figure S3: Components and steps used to fabricate the microfluidic device. A) The US transducer; B) the 3D printed transducer mold; C) the 3D printed alignment frame; D) the CNC machined metal mold. (E) The set of molds with cured PDMS. F) The transducer mold replaced by the US transducer in the final microfluidic device. G) The backside view of the device.

Flow diagram of the flow system

The acoustic flow cytometer contains four distinct modules, namely fluid, RF, trigger, and optical (Fig. S4). The fluid module consists of the microfluidic device, tubing, and pumps. The hydrodynamically

focused sample flow passes through an interrogation zone inside a microfluidic channel. The individual cells or particles in the sample flow are individually interrogated by both US and laser pulses, sequentially.

The RF module accounts for all the components that are used for emitting ultrasound (US) and detecting, and recording both US and photoacoustic (PA) signals. The tools that generate, synchronize, and control the triggers are grouped as a trigger module. The microfluidic device together with the pumps are grouped as a fluid module. Finally, all the optical systems, including lasers, optical components, and camera, are in the optical module.

A US transducer is integrated with a microfluidic device. This transducer switches between emission and receiving modes. During the emission mode, a US pulse is emitted towards the sample particles, and during the receiving mode, it detects the US signals from the particles. The modes are controlled by a trigger-controlled coupler (Mini-circuits, Brooklyn, NY, USA), which switches the connection of the transducer with a pulse generator and an amplifier for emission and receiving modes, respectively. The detected signals from the transducer are amplified by an amplifier and are recorded by a digitizer for post-processing. The complete process is shown as an RF module in the flowchart of Fig. S4.

The synchronization of the components in the system is maintained by various trigger signals. A control trigger operating at 4.1 KHz is generated by a PCI card and triggers a pulsed laser source. This complete process flow is shown as Trigger module in the Fig. S4. Each laser pulse generates a TTL signal, which is synchronized with three other signals (served as triggers for pulser, switch, and digitizer) generated by two function generators connected in series (Fig. S4). Both function generators have an internal delay of 250 ns. As a result, US backscatter detected by the US transducer is delayed. This synchronization problem can be avoided by using an external TTL switch that branches to a single input to three branches of synced TTL signals. In addition to the internal delay due to the function generators, we added a 250 ns delay time to the trigger to prevent PA signals from being coincident in time with the US transducer artifacts[1], which are orders of magnitude stronger than the PA detected from the cells or particles.

Simultaneously, laser pulses are also focused on the particles by a 10X optical objective. The laser pulses generated from a laser source transmits through an optical fiber and multiple reflecting and dichroic mirrors before being focused by the objective. A camera is connected to observe the microfluidic channel in real-time.

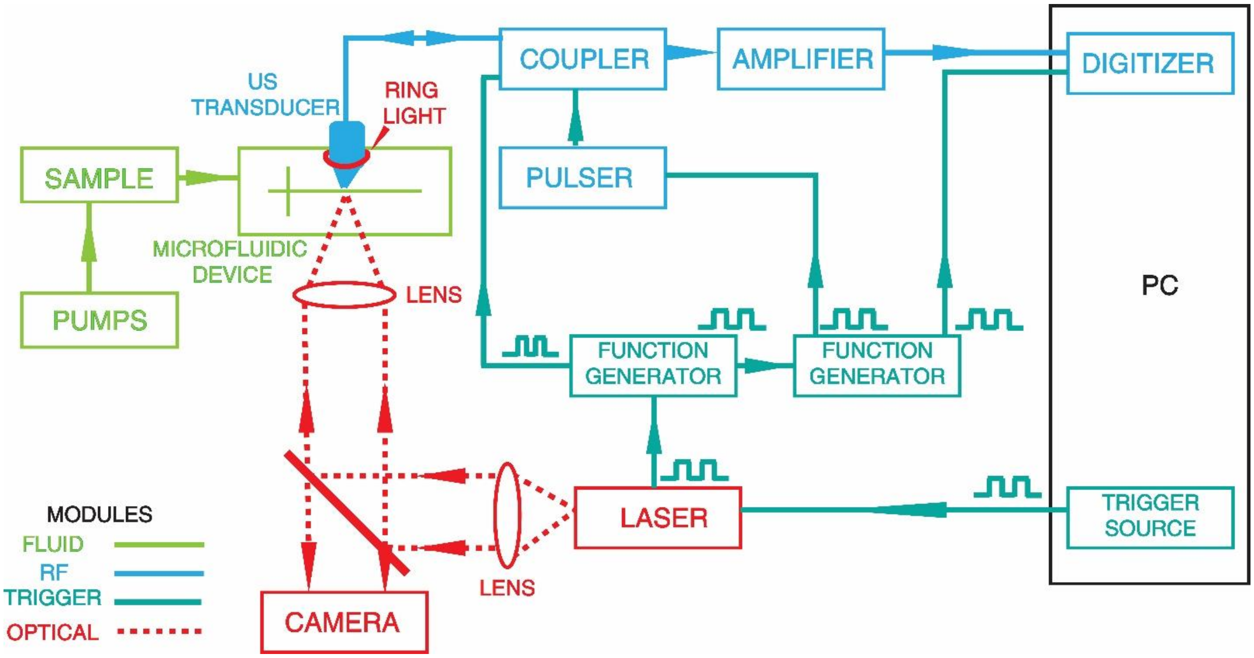

Figure S4: Flow diagram of the acoustic flow cytometer. Arrows indicate the direction of travel of either signals, fluids, or light depending on the module.

References:

1. Briggs, A. & Kolosov, O. *Acoustic microscopy*. (Oxford University Press, 2009).
